# Medical Society of the County of Erie

**Published:** 1902-02

**Authors:** Franklin C. Gram

**Affiliations:** Secretary


### Medical Society of the County of Erie.

*Eighty-first Annual Meeting.*

Reported by FRANKLIN C. GRAM, M. D., Secretary.

THE eighty-first annual meeting of the Medical Society of the County of Erie was convened at 10.20 a.m., January 14, 1902, by Dr. Wm. C. Phelps, president, in the parlors of the Y.M.C.A., Buffalo.

The minutes of the previous meeting were read and approved.

Dr. William Warren Potter, chairman of the committee on membership, presented the name of Dr. H. Arnold Pierce, of Blasdell, and recommended his election to membership. His recommendation was adopted.

President Phelps called vice-president Ward to the chair and then delivered his annual address, in which he briefly reviewed the history of the society and made certain important recommendations. (See page 486.)

Librarian Callanan asked permission to have the books of the society, now in the library of the University of Buffalo, indexed by the librarian of that institution, and also that he be permitted to issue a circular to members requesting donations of medical works published during the past century. His requests were granted.

Chairman COAKLEY, of the Board of Censors, submitted the following report:

BUFFALO, N.Y., January 9, 1902.

*To the Medical Society of the County of Erie:*

*Gentlemen*--Your board of censors respectfully reports that since its last report made to the Society in June, 1901, the censors have continued their work of investigating cases of alleged illegal practitioners of medicine and surgery.

During the past half year complaints have been lodged against numerous persons engaged in business and advertising themselves as Dr. so and so, palmist, magnetic healer and other descriptions of a like character. Whenever a complaint has been made to your board in reference to any party thus advertising, investigation has been made and wherever it was found that the law was being violated the parties have been warned, and in every instance your board believes that the parties so engaged in violation of the law have left the city or ceased such violation.

There has also been a complaint lodged with the board of censors against William H. Barrett, who was advertising himself as "Dr. William H. Barrett, eye specialist, etc." Upon investigation, it was found that Mr. Barrett was unlawfully advertising himself as "Doctor" and upon being warned he at once discontinued the use of such title in advertising. Several other instances of parties claiming to be specialists in some particular line have been reported to your board as illegally using the title "Dr." in their advertising matter, upon signs, etc., and in every instance your board has warned the parties so violating the statute, and upon such warning being given the illegal signs have been taken down and illegal advertising discontinued.

Perhaps the most important case since our last semiannual report is that of Prof. A. J. Wiechers, who advertised himself as "Antonius, the boy phenomenon," with offices at No. 685 Main Street.

In the early part of December, 1901, complaints were made to your board by several parties who had been induced to receive treatment from Prof. Wiechers and his alleged staff of physicians, that said professor and his assistants were illegally practising medicine, and the chairman of your board upon investigation learned that such complaints were well founded and visited the offices of Prof. Wiechers and his staff, at 685 Main Street, and warned them that they must either comply with the statutes of the State of New York or discontinue such practice of medicine. It appearing that such warning was unheeded the chairman of your board thereafter caused warrants to be issued for the arrest of Professor Wiechers and the alleged consulting physicians, J. H. McCaffrey, William Rear, Otto Urban and A. L. Salter.

The police officers were unable to find and serve the warrants issued against Otto J. Urban and William Rear, said parties having apparently left the city, or being merely fictitious names, used for the purpose of duping the public by the "Boy Wonder Antonius."

Upon the hearing before Judge Murphy of the Police Court, it appeared that A. L. Salter was duly registered as a physician and surgeon under a slightly different name and he was discharged. It also appeared that J. H. McCaffrey had, subsequent to the warning given by the chairman of your Board of Censors, complied with the statute and had registered himself as a physician and surgeon, and upon such fact appearing, your chairman permitted the proceeding against him to be discontinued.

Prof. A. J. Wiechers, otherwise known as Antonius the Boy Phenomenon, was held by Judge Murphy for the Grand Jury under bail of $200. Your chairman has since consulted with the District Attorney of Erie County in reference to the case, and the matter will be presented to the Grand Jury on the 10th day of January, 1902, or as soon thereafter as the business of the District's Attorney's office will permit, and your board of censors confidently expects that an indictment will be found against him.

In conclusion this board respectfully recommends that the Hon. Tracy C. Becker be continued as counsel for the board as heretofore, and that this board be given authority to employ such detectives and other agents as may be required for the performance of its duties, and to draw upon the treasury as before for their reasonable compensation.

All of which is respectfully submitted.

J. B. Coakley, *Chairman.*<br>

H. R. Hopkins.<br>

Francis E. Fronczak.<br>

Irving W. Potter.

On motion the last paragraph was so changed as to permit the board to employ any attorney at its discretion, and the entire report was then adopted.

The president appointed Drs. Wm. C. Krauss, Abbott and Clements a committee on nominations. They submitted the following nominations, who were all duly elected: President, Walden M. Ward, North Collins; vice-president, Ernest Wende; secretary, Franklin C. Gram; treasurer, Edward Clark; librarian, Wm. C. Callanan; censors, J. B. Coakley, chairman; H. R. Hopkins, Henry C. Lapp, F. E. Fronczak, I. W. Potter.

Committee on legislation: Ernest Wende, William Warren Potter and Edward Clark.

Additional delegates to the Medical Society of the State of New York: C. A. Clements, W. D. Bidaman, J. J. Twohey, E. M. Dooley, B. S. Bourne, Wm. G. Taylor.

Delegates to the Medical Association of Central New York: Drs. Thomas G. Allen, B. Bartow, Geo. L. Brown, Geo. F. Cott, C. C. Frederick, F. W. Hinkel, H. R. Hopkins, S. Y. Howell, Max Keiser, M. D. Mann, J. W. Putnam, DeLancey Rochester, G. W. York and S. H. Lynde.

Treasurer CLARK submitted his annual report which, with the books and vouchers, was referred to Drs. Potter, Callanan and Walsh for audit. The committee reported finding them correct.

Dr. JOHN H. GRANT presented the following resolution:

*Resolved*, By the Medical Society of the County of Erie, State of New York, that the committee on legislation of the Medical Society of the State of New York, be requested to urge and aid the passage in the Assembly and Senate, the bill of the Hon. Mr. Nye, amending the public health laws by excepting regularly licensed and practising physicians of this state from the prohibitory clause which prevents said physicians from conducting a drug store, as the preparation of medicines, as well as their administration comes within the privileges of the practice of medicine.

*Resolved*, That the delegates of this society accredited to the Medical Society of the State of New York are requested to aid by all proper means the passage of said bill by securing the coöperation of the state society to that end.

The resolution was adopted.

Dr. C. E. CONGDON made a brief verbal explanation regarding the work of his special committee.

Dr. A. L. BENEDICT offered the following resolution, which was adopted:

*Resolved*, That the Medical Society of the County of Erie memorialize the Medical Society of the State of New York, requesting such action by the latter, at its stated meeting in the current month, as may lead to representation in the American Medical Association, of the Medical Society of the State of New York.

*Resolved*, That, in like manner, the Medical Society of the County of Erie express its earnest desire for the unity of the regular medical profession of the State of New York.

Dr. ERNEST WENDE, former health commissioner of Buffalo, read a paper on the Diagnosis of Smallpox, and Dr. Walter D. Greene, health commissioner of Buffalo, gave a very interesting statement relative to the present epidemic of smallpox here and elsewhere. (See page 492.)

These papers elicited some very interesting discussions, participated in by Drs. Wetmore, George Abbott, O'Brien, Krauss, S. S. Greene and Pettit.

Dr. WM. C. KRAUSS introduced the following resolution, seconded by Dr. S. S. Greene and unanimously carried.

*Resolved*, That in view of the difficulties encountered and the great expense incurred in the care, treatment and isolation of smallpox cases and in further view of the important bearing on health and life it is the sense of this meeting that a new modern isolation hospital is of immediate necessity, and furthermore, that we pledge our best efforts towards accomplishing such results.

Dr. GEO. F. COTT read a paper on Sinus thrombosis following acute sore throat.

A vote of thanks was tendered the essayists and all the officers, after which the society adjourned.

